# Torsional phacoemulsification: A pilot study to revise the “harm scale” evaluating the endothelial damage and the visual acuity after cataract surgery

**DOI:** 10.1371/journal.pone.0186975

**Published:** 2017-10-26

**Authors:** Francesco Saverio Sorrentino, Silvia Matteini, Aurelio Imburgia, Claudio Bonifazzi, Adolfo Sebastiani, Francesco Parmeggiani

**Affiliations:** 1 Department of Surgical Sciences, Unit of Ophthalmology, Ospedale Maggiore, Bologna, Italy; 2 Department of Biomedical and Surgical Sciences, Division of Ophthalmology, University of Ferrara, Ferrara, Italy; 3 Department of Surgical Sciences, Unit of Ophthalmology, Ospedale di San Marino, San Marino, Repubblica di San Marino; 4 Department of Biomedical and Surgical Sciences, Section of Human Physiology, University of Ferrara, Ferrara, Italy; 5 Clinic of Ophthalmology, University of Ferrara, Ferrara, Italy; University of Illinois at Chicago, UNITED STATES

## Abstract

**Purpose:**

To study the effect of torsional phacoemulsification energy on corneal endothelium evaluating the relationship between changes of endothelial cells and postoperative visual acuity.

**Methods:**

This prospective clinical observational cohort study included 50 patients with cataract who underwent torsional phacoemulsification. Sequential quantitative and qualitative morphometric endothelial cell analyses of the cornea were performed four weeks preoperatively and six weeks postoperatively using noncontact specular microscopy.

**Results:**

This work confirmed the strong relationship, described by a linear model (one-way ANOVA, R^2^ = 77.9%, P < 0.0001), between the percentage of endothelial cell loss (ECL%) and the 5-score harm scale. According to the Tukey post-hoc pairwise comparison test, distinct values of ECL% are grouped in 3 subsets. The value of ECL = 10% has been identified as cut-off to discriminate patients with excellent postoperative best-corrected visual acuity (BCVA > 85 letters) from those with just a good/satisfied visual outcome (BCVA ≤ 85 letters). Within the 5-score harm scale, there was a significant correlation among phaco energy intraoperatively delivered and the average endothelial cell loss.

**Conclusions:**

This study confirms the validity of the 5-score harm scale first proposed by Sorrentino and colleagues in 2016. This time, the method categorizes cataracts taking into account nucleus hardness and phaco cumulative dissipated energy. Predicting the harm on corneal endothelium, we can discriminate patients with excellent BCVA and with just good/satisfied BCVA. With torsional phacoemulsification with respect to longitudinal, the percentage of patients who can reach excellent BCVA is remarkably increased.

## Introduction

In last years, novel techniques, new machines and helpful viscoelastic devices have allowed cataract surgery to be safer and faster [1]. The integrity of the capsular bag and the care of corneal endothelium have always been the main concerns of surgeons. Several factors contribute to bring about stress on corneal endothelium such as ultrasonic power fluctuation, bouncing of fragments, fluid turbulence and increased production of free radical oxygen species [2–4].

Unlike the longitudinal mode using the conventional jackhammer effect of the phaco tip, the torsional mode works through rotary oscillations of the phaco tip reducing the ultrasound power and limiting the shearing forces of breaking up cataracts [5–6]. In previous studies, uneventful cataract surgery has been observed to induce endothelial cell loss ranging from 12% to 20% [7–9]. Most of recent literature discuss about the femtosecond laser-assisted cataract surgery as minimally invasive procedure towards corneal endothelium, but we would like to pay much more attention to traditional phacoemulsification and its effect on corneal endothelial cells.

The corneal endothelium is a single layer of hexagonal cells that lines the posterior corneal surface and cannot proliferate because cells are stuck in the G1 phase of the cell cycle [10]. During life, the corneal endothelial cell density gradually decreases. In case of harm to endothelium, for instance after cataract surgery, the surviving endothelial cells (ECs) spread out, changing their size and shape over the time [11,12]. The healthy endothelium is characterized by a sort of “pump-leak” function, which enables to maintain corneal transparency and well-balanced stromal hydration [13]. Routinely, we use non-contact specular and confocal microscopy to clinically investigate and to analytically evaluate the corneal endothelium [14,15].

The aim of this study was to investigate the effect of energy delivered into the anterior chamber following torsional phacoemulsification. We have used statistical criteria to analyze changes on ECs and the effects of phaco cumulative dissipated energy (CDE) on corneal endothelium. We used the “harm scale”, the method we had proposed in 2016, to study alterations and damage on ECs [9]. Then, we investigated the relationship between loss of ECs and postoperative visual acuity.

## Materials and methods

This prospective longitudinal clinical study included a total of 50 eyes of 50 patients and was conducted from January 2016 to September 2016 at Ferrara University Hospital. The tenets of the Declaration of Helsinki were followed. The study was in compliance with the Health Insurance Portability and Accountability Act (HIPAA) requirements and was approved by the Institutional Review Board of the Azienda Ospedaliero-Universitaria ‘‘S. Anna”, Ferrara, Italy. The Ethics Commette of the Univeristy of Ferrara approved this study.

The number of 50 patients with age-related cataract was considered as representative sample of a population that reflects the features of people affected by uncomplicated cataract. Patients diagnosed with age-related cataract underwent surgery, after being provided with written informed consent. Following the same protocol we did in our previous study about longitudinal phacoemulsification, we chose to perform the standard ophthalmic examination four weeks before surgery and to schedule patients to be seen at one day, five days and six weeks after surgery [9]. The exclusion criteria were as follows: pseudoexfoliation, diabetes, corneal distrophies, ocular trauma, glaucoma, optic neuropathies, uveitis, high-degree myopia (axial length longer than 26 mm), high-degree hyperopia (axial length shorter than 21 mm), previous ocular surgery.

We applied to this investigation the same protocol we had used in the first pilot study we did about longitudinal phacoemulsification, as already described in detail in the section “Materials and Methods” in the work of Sorrentino and coworkers in 2016 [9].

During the general eye examination, at slit lamp, the nucleus density grade was evaluated according to the Lens Opacities Classification System II (LOCS II) [16]. The non-contact specular microscopy was performed with EM-3000 (Tomey GmbH, Erlangen, D) four weeks before and six weeks after surgery, in order to measure the endothelial cell density (ECD) and other features of ECs, and to determine the surgically-induced endothelial cell loss (ECL: difference between preoperative ECD and postoperative ECD). The device is equipped with an autofocus, digital image-capturing system, and automated image analysis software. A fixed area of 0.135 mm^2^ (0.25x0.54 mm) allows for counting up to 300 cells per image. An automated cell contour recognition algorithm, based on contrast differences and area-based counting technique, is used to acquire measurements, with internal calibration for magnification [17]. The phaco machine was the Infiniti System (Alcon Laboratories, Inc, Fort Worth, Tex, US). The phacoemulsification energy through the tip was set up with torsional energy mode. The best-corrected visual acuity (BCVA) was measured at 6-week follow-up postoperatively, using the 4 meter 2000 series revised ETDRS Chart (Precision Vision). Intraocular lens power was calculated with Sanders-Retzlaff-Kraff/T formula using preoperative keratometry and axial length measurements acquired with IOLMaster 500 (Carl Zeiss Meditec AG, Jena, D). Experienced surgeons did cataract surgery performing the divide and conquer nucleofractis technique. In order to protect the endothelium, surgeons used discovisc (Alcon Laboratories, Inc, Fort Worth, Tex, US) as ophthalmic viscosurgical device and initially performed hydrodissection of the lens cortex and hydrodelineation of the nucleus [18]. A preloaded hydrophilic single-piece intraocular lens was implanted in the capsular bag for each patient. No suture of the temporal corneal wound was required. There were no significant intraoperative complications such as posterior capsule tear, vitreous loss, capsular bag dislocation, or aphakia. Also, there were no significant postoperative complications such as endophthalmitis or severe intraocular inflammation. We used dexamethasone 0.1% eyedrops four times per day for 15 days and bromfenac two times per day for 15 days to control postoperative inflammation.

Intraoperatively, we registered the phaco CDE after phase 1 and 2. CDE#1 was the CDE for sculpting and cracking of nucleus, CDE#2 was the total CDE used for phacoemulsification (sculpting, cracking, fragmentation and aspiration of quadrants). The continuous mode is a type of power modulation enabling linear control of phaco and aspiration by foot pedal [19,20]. The maximum torsional ultrasound power and vacuum for each phase were preset by surgeons. In our study, CDE#1 ranged from 1.15 to 33.25, while CDE#2 ranged from 6.83 to 55.36.

We have applied the “harm scale”, featured by 1 to 5 score, to describe morphometrical damages and loss of ECs after torsional phacoemulsification [9]. Three parameters have been taken into consideration to construct this Likert-type scale: the grade of cataract hardness, evaluated according to the LOCS II, the CDE of phaco phase 1 (CDE#1) and the whole CDE of phacoemulsification (CDE#2) [16]. We pursued two goals using the 5-score harm scale: prediction of ECL% and discrimination between excellent and good/satisfied postoperative BCVA. All data we collected pre-operatively, intraoperatively and post-operatively can be found in Supplemental Files (S1 File).

Data were analyzed with the MINITAB software (MINITAB Inc., Pennsylvania State College, USA). A descriptive analysis was performed and the normality test of the data was carried out. The *t*-Student test was used to compare the results between two groups and variance analysis (ANOVA) was used to compare the five levels of the endothelial cell damage. The value of P < 0.05 was considered for statistical significance.

## Results

**Fig 1:**
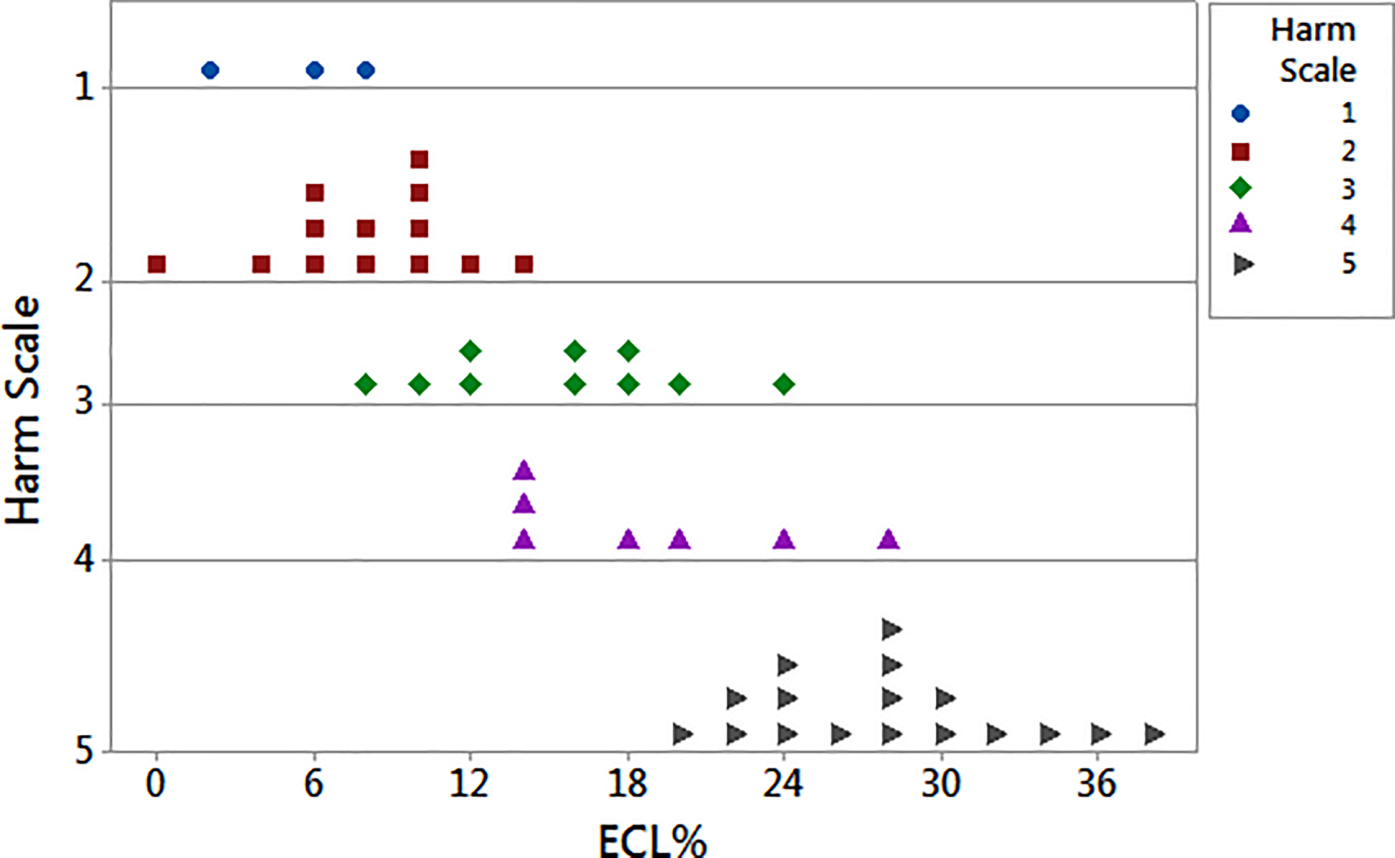
Dotplot of distribution of endothelial cell loss percent depending on the 5-score harm scale. There are 3 patients in level 1 (blu circle dots), 13 in 2 (red square dots), 10 in 3 (green rhombus dots), 7 in 4 (purple triangle dots), and 17 in 5 (grey right arrow dots). ECL = endothelial cell loss.

The dotplot chart of Fig 1 showed the relationship between ECL% and each score of the harm scale. The distribution of dots seemed to be consistent from low scores (only 3 dots in score 1) to high scores (about one third of dots in the only score 5). The distinct levels are partly overlapped. The first two groups (score 1 and 2), made up of *soft* cataracts, corresponded to ECL% underneath 10% and could be even merged.

The dots of levels 3 and 4 were grouped, somewhat, around intermediate values of ECL% (12–24%). The 5-score dots were grouped, with very little dispersion, around high values of ECL% (24–36%), representing very *hard* cataracts.

**Fig 2:**
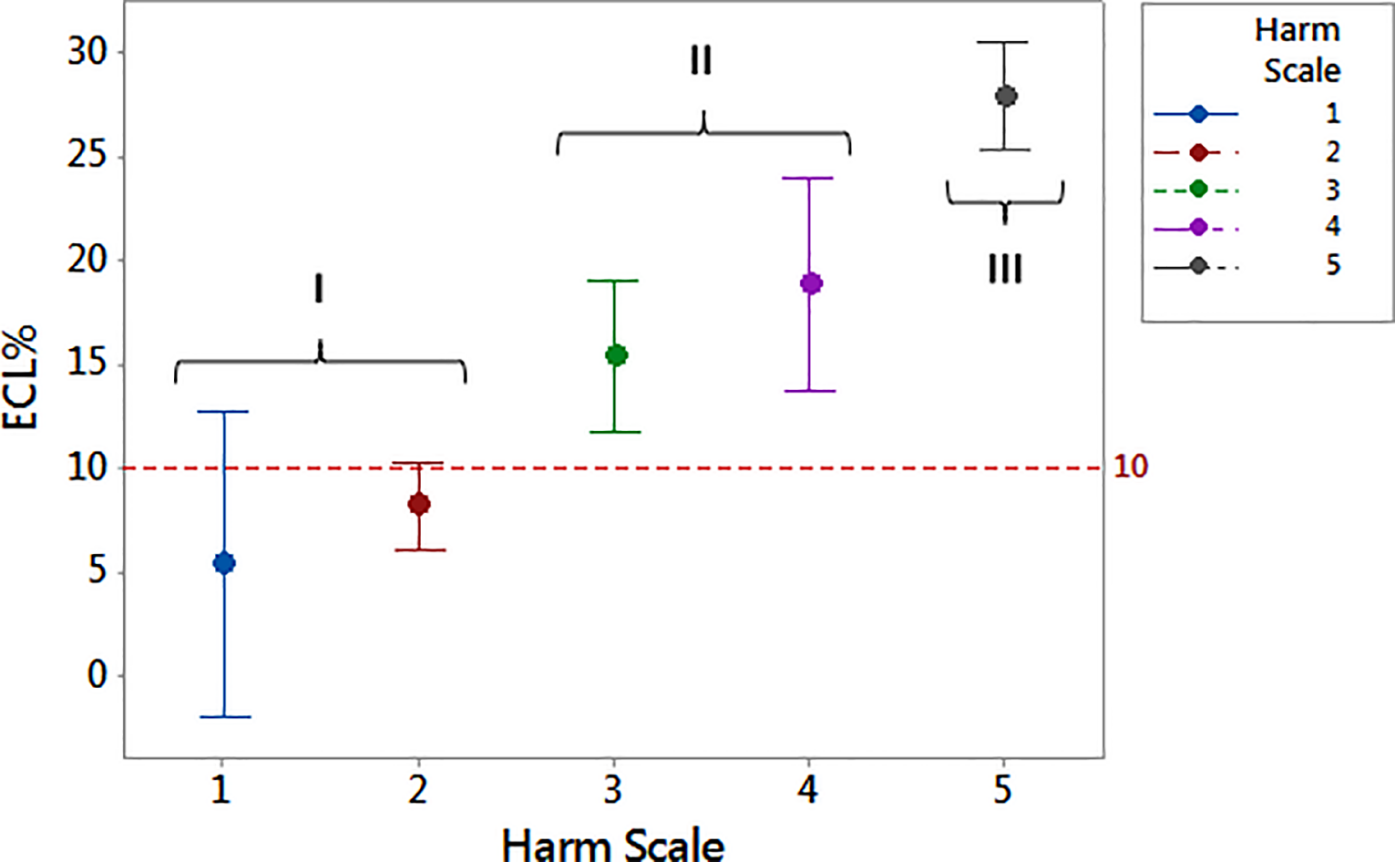
Interval plots of endothelial cell loss resulting from one-way ANOVA test for increasing score values. Tukey post-hoc test groups score 1–2 into subset I, score 3–4 into subset II and score 5 into subset III. The cut-off ECL = 10% splits into low scores (subset I, 28% of patients) and high scores (subsets II and III). ECL = endothelial cell loss.

The analysis of variance (one-way ANOVA, P < 0.0001) showed that in the harm scale values of ECL% were grouped according to their dispersion as stated from the partially overlapping interval plots 95% confidence interval (CI) (Tukey post-hoc test). The Fig 2 displayed the rising trend of ECL% as the score of the harm scale gradually increases (R^2^ = 77.9%). The three subgroups (I, II, III) resulting from Tukey post-hoc test confirmed the distribution of ECL% for rising score (Fig 1). Except for score 1 and partly for score 4, each interval plot 95% CI is rather small so as to underline the little dispersion of our values for distinct scores (Fig 2). We chose the value of ECL% = 10% as cut-off between low scores (subset I) and high scores (subsets II and III) because it is higher than the mean value of ECL% for score 1 and 2 (subset I) of the harm scale.

**Fig 3:**
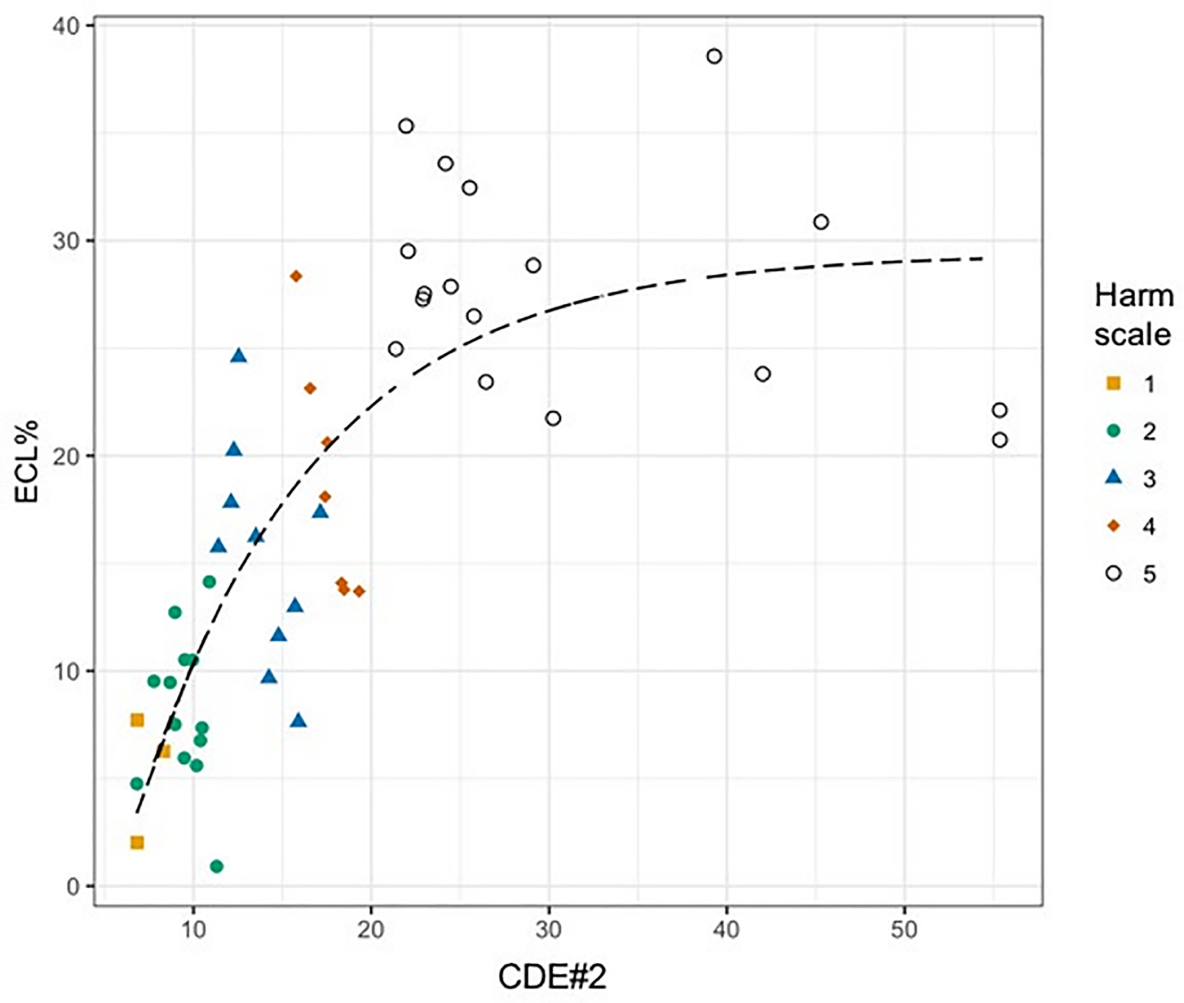
Scatterplot of distribution of percentage of endothelial cell loss depending on the total phaco cumulative dissipated energy, according to the 5-score harm scale. The non-linear curve well describes the rising trend of endothelial cell loss for increasing cumulative dissipated energy till a fixed level where a maximum ECL% is achieved. Dots (score 1–4) are all grouped along the first linear growth. Only dots of score 5 are uniformly distributed around the final asymptote. CDE#2 = total phaco cumulative dissipated energy; ECL = endothelial cell loss.

We also used the harm scale to describe the entire energy delivered into the anterior chamber after torsional phacoemulsification. The Fig 3 showed the distribution of ECL% depending on the global CDE (CDE#2) at the end of the cataract surgery. This correlation was well-described by a non-linear curve, asymptotically reaching ECL% of about 30%. The first linear growth, which approximately went up to 20–25% of ECL%, was linked to the interval from 0 to 20 of CDE#2 featured by the most incidence of harm on endothelial cells. This interval included more than 60% of tested sample. The asymptote corresponded to values of ECL% above 25% and CDE#2 higher than 20. This interval collected patients with hard cataract and the highest score of the harm scale. Considering the 5 scores of the harm scale and the results of Tukey post-hoc test (3 subsets), we could see in Fig 3 the peculiar distribution of our subgroups along the curve: subgroup I and II stood on the first steep line, whereas subgroup III stood on the second flat line. The curve of Fig 3 resulted from the least square analysis approach as *ECL*% = *a* + (*b* − *a*) * exp[−exp(*c* * *CDE*#2)].

After describing the rising trend of ECL% depending on CDE#2, we analyzed two groups of patients (A, B) on the strength of the cut-off value ECL = 10% as above stated by one-way ANOVA test (Fig 2). We chose this cut-off value to discriminate patients who were likely to have an excellent recovery of visual acuity after surgery (more than 85 letters of ETDRS) and patients who were going to have a good/satisfying visual outcome (85 letters of ETDRS or less). The group A was characterized by ECL less than 10%, whereas the group B included patients with ECL equal to or more than 10%. In our study, we found more patients in group B (36 people, 70% of sample) than A (14 people, 30% of sample).

**Fig 4:**
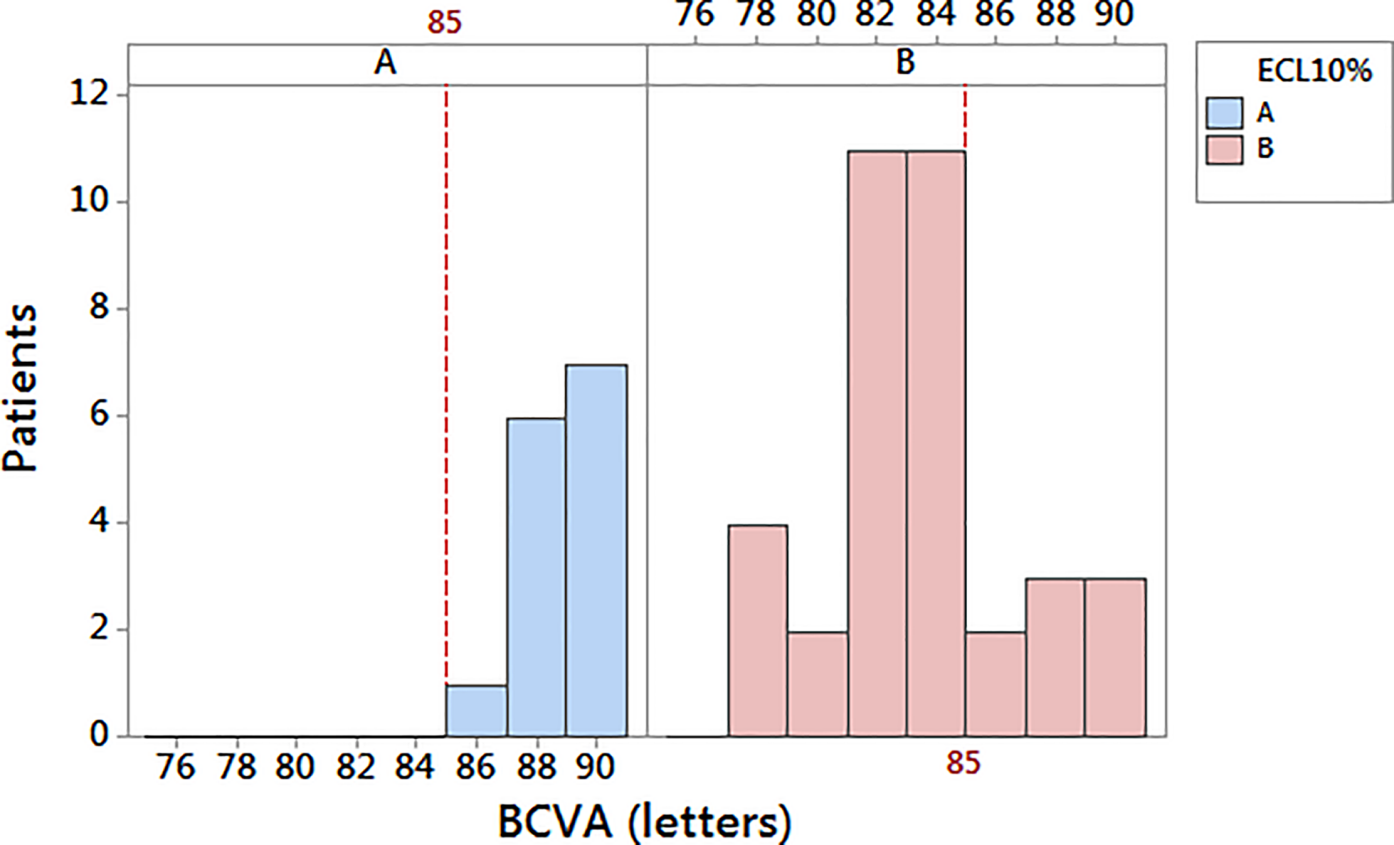
**Frequency distribution of postoperative best-corrected visual acuity in group A (ECL < 10%) and B (ECL ≥ 10%) at 6-week follow-up.** Line at 85 letters discriminates an excellent visual outcome (group A) from a good/satisfying visual function (group B). BCVA = best-corrected visual acuity; ECL = endothelial cell loss.

Observing different ECL% in groups A and B for increasing score values, we compared the BCVAs of patients in both groups, evaluating how the loss of endothelial cells due to torsional phacoemulsification could affect the visual acuity at 6-week follow-up. The histogram in Fig 4 displayed the comparison between BCVA of group A (ECL < 10%) and group B (ECL ≥ 10%). Though both groups had good recovery of visual acuity, there was an interesting difference in the distribution of BCVA in these two groups. Fixing a cut-off at 85 letters, which meant quite a good visual outcome after phacoemulsification, we could see that 100% of people in group A had BCVA more than 85 letters at ETDRS chart (excellent visual acuity). Also, in group B approximately 22% of subjects had excellent BCVA (more than 85 letters), while about 78% had good/satisfying BCVA (equal or less than 85 letters) postoperatively.

## Discussion

In this study we revised the “harm scale”, the new method that we had already constructed to categorize distinct cataracts in order to try and predict the postoperative visual acuity. As we had prefixed, we had two end-points: first to make a connection between any type of cataract and the harm on corneal endothelium, secondly to establish a relationship between the loss of endothelial cells and the postoperative visual outcome. This time, we have applied the harm scale to torsional phacoemulsification, thus we included an important parameter in the construction of the scale: the cumulative dissipative energy (CDE) of phacoemulsification that the phaco machine automatically registers itself. Therefore, the three variables implied, all intraoperatively acquired, were the hardness grading, obtained according to LOCS II, the CDE of phaco phase 1 (CDE#1) and the whole CDE of phacoemulsification (CDE#2).

As explained in our previous work, ours is a Likert-type scale giving a score from 1 to 5 to each cataract (Table 1) [9]. The name of “harm scale” was meant to highlight changes and/or injures on corneal endothelium after cataract surgery [21–24]. We still maintained the distinction in five levels in order to compare, in the next future with larger samples, the harm scale for longitudinal to torsional phacoemulsification.

**Table 1:** The construction of the harm scale.

Score of Likert-type harm scale	1	2	3	4	5
Hardness	1	1–2	2–3	3–4	4
CDE#1	<2.5	<5	<7.5	<10	≥10
CDE#2	<5	<10	<15	<20	≥20
ECL% Mean	5.3	8.1	15.4	18.8	27.9
ECL% SE	1.7	1.0	1.6	2.1	1.2
Number of patients	3	13	10	7	17

CDE#1, phaco “metrics” to do sculpting; CDE#2, phaco “metrics” to do the entire phacoemulsification; ECL, endothelial cell loss; SE, standard error; AVG, average cell area. The upper part shows the method of construction of the 5-score harm scale by collecting three parameters (hardness grading, CDE#1 and CDE#2). Hardness grading is according to Lens Opacities Classification System II; CDE#1 and CDE#2 are the cumulative dissipated energies automatically registered by phaco machine. The bottom part displays the rising trends of endothelial cell loss for increasing scores; ECL% standard errors give indications of the accuracy of such analysis.

Once again, we confirmed that hardness grading is a preliminary approximate evaluation of any cataract just providing vague indications for surgical procedure. By the way, doing surgery as well as managing complications, especially for cataracts more complex than expected, is characterized by a sort of variability intraoperatively. So the harm scale could be considered as refinement of the initial assessment of hardness, also comprising intraoperative CDE phaco, remarkable variable directly visualized on the screen of the machine at the end of surgery.

Table 2 sums up our results displaying the rising trend of ECL% for increasing scores of the harm scale. The five levels could be grouped in 3 subsets as certified by Tukey post-hoc test (ANOVA, P < 0.001): grouping I for score 1 and 2, grouping II for score 3 and 4, grouping III for score 5. This analysis allows us to establish the cut-off of ECL% = 10% to discriminate low scores (subset I) from high scores (subsets II and III). From this result, we obtained two ECL groups of patients (A for subset I, B for subsets II and III), which have been remarkable to grasp the slight differences in postoperative BCVA. In fact, we realized that patients having ECL less than 10% could get an excellent visual recovery measured as postoperative BCVA > 85 letters, whereas patients with ECL greater or equal 10% could get a good/satisfying visual acuity postoperatively featured with BCVA ≤ 85 letters.

**Table 2:** An overview of the 5-score harm scale: Significant correlations.

Score	Patients	Post-Hoc Tukey Groups	ECL% Mean	BCVA > 85 Letters	BCVA ≤ 85 Letters
1	3	I	5.3	3	0
2	13	I	8.1	13	0
3	10	II	15.4	4	6
4	7	II	18.8	1	6
5	17	III	27.9	1	16

ECL, endothelial cell loss; BCVA, best-corrected visual acuity. Mean values of percentage of endothelial cell loss related to the 5-score harm scale as results from post-hoc Tukey test. The last two columns highlight the number of patients who obtained an excellent (BCVA > 85 letters) and a good/satisfying best-corrected visual acuity (BCVA ≤ 85 letters) postoperatively.

This study has been carried out with a sample of 50 patients. We did not enroll other people because the relationship between ECL% and the total CDE seemed to be well established. In future, we would like to enlarge the sample size in order to fine-tune the harm scale diminishing the dispersion of some values. In this pilot study, we applied our method to torsional phacoemulsification seeking for a connection between the rising trend of ECL% and postoperative BCVA for increasing scores of the harm scale. Of course, further studies are needed to better manage this innovative method of analysis aiming to a challenging prevision model linking the ECL% and CDE delivered in the anterior chamber.

In conclusion, the harm scale is a very good strategy to estimate the damage on corneal endothelium after torsional phacoemulsification. The striking relationship between loss of corneal endothelial cells and postoperative visual outcome is remarkable, once again suggesting to perform the best surgical procedure and pay attention, at all times, to maintain as low level of CDE as it is possible.

## Supporting information

S1 File Data used to construct the new version of the harm scale after torsional phacoemulsification. (XLSX)
